# A Review of Seafood Safety after the *Deepwater Horizon* Blowout

**DOI:** 10.1289/ehp.1103507

**Published:** 2011-05-12

**Authors:** Julia M Gohlke, Dzigbodi Doke, Meghan Tipre, Mark Leader, Timothy Fitzgerald

**Affiliations:** 1 Department of Environmental Health, and; 2 Department of Epidemiology, School of Public Health, University of Alabama at Birmingham, Birmingham, Alabama, USA; 3 Environmental Defense Fund, Washington, District of Columbia, USA

**Keywords:** *Deepwater Horizon*, dispersants, Gulf of Mexico, heavy metals, oil spill, polycyclic aromatic hydrocarbons, risk assessment, seafood

## Abstract

Background: The *Deepwater Horizon* (DH) blowout resulted in fisheries closings across the Gulf of Mexico. Federal agencies, in collaboration with impacted Gulf states, developed a protocol to determine when it is safe to reopen fisheries based on sensory and chemical analyses of seafood. All federal waters have been reopened, yet concerns have been raised regarding the robustness of the protocol to identify all potential harmful exposures and protect the most sensitive populations.

Objectives: We aimed to assess this protocol based on comparisons with previous oil spills, published testing results, and current knowledge regarding chemicals released during the DH oil spill.

Methods: We performed a comprehensive review of relevant scientific journal articles and government documents concerning seafood contamination and oil spills and consulted with academic and government experts.

Results: Protocols to evaluate seafood safety before reopening fisheries have relied on risk assessment of health impacts from polycyclic aromatic hydrocarbon (PAH) exposures, but metal contamination may also be a concern. Assumptions used to determine levels of concern (LOCs) after oil spills have not been consistent across risk assessments performed after oil spills. Chemical testing results after the DH oil spill suggest PAH levels are at or below levels reported after previous oil spills, and well below LOCs, even when more conservative parameters are used to estimate risk.

Conclusions: We recommend use of a range of plausible risk parameters to set bounds around LOCs, comparisons of post-spill measurements with baseline levels, and the development and implementation of long-term monitoring strategies for metals as well as PAHs and dispersant components. In addition, the methods, results, and uncertainties associated with estimating seafood safety after oil spills should be communicated in a transparent and timely manner, and stakeholders should be actively involved in developing a long-term monitoring strategy.

Current estimates suggest that the *Deepwater Horizon* (DH) blowout resulted in the release of approximately 4.4 million barrels ± 20% (7.0 × 10^5^ m^3^) into the northern Gulf of Mexico over a 3-month period during the summer of 2010 (Crone and Tolstoy 2010). The leak was a result of a deepwater rig explosion on 20 April 2010 due to methane gas release after drilling an exploratory well. An attempt to activate a safety feature to prevent a blowout failed. After burning for 36 hr, the entire platform sank to the seafloor.

Because of concerns over seafood safety, on 2 May 2010, the National Oceanic and Atmospheric Administration (NOAA) initiated closures of federal waters to commercial and recreational fishing; Louisiana, Mississippi, Alabama, and eventually Florida subsequently instituted fisheries closures in state waters, in coordination with the U.S. Food and Drug Administration (FDA) (Figure 1). By 21 June, closures covered approximately 37% of the Gulf of Mexico (225,290 km^2^), extending east from Atchafalaya Bay, Louisiana, to Panama City, Florida (NOAA 2010b). The well was capped on 15 July, and on 19 September, relief wells were completed that permanently disabled the well. Reopening of closed areas to fishing began on 23 June, although NOAA reclosed 10,911 km^2^ to deepwater fishing for royal red shrimp northwest of the wellhead on 24 November 2010 (NOAA 2010a). On 21 January 2011, only 0.4% of federal waters (1,041 mi^2^; 2,697 km^2^) immediately surrounding the well remained closed to fisheries. As of 19 April 2011 all Gulf of Mexico federal waters are open to fisheries.

**Figure 1:**
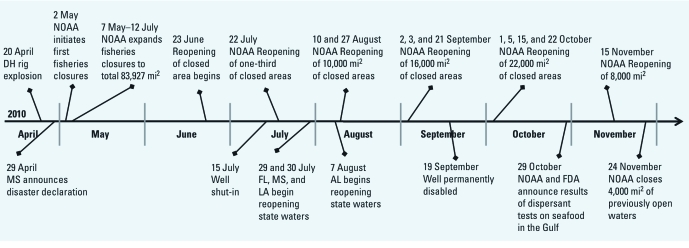
Timeline of fisheries closures and reopenings in the Gulf of Mexico due to the DH blowout in 2010. Abbreviations: AL, Alabama; MS, Mississippi; LA, Louisiana; FL, Florida. Data from NOAA (2010b) and FDA (2010). On 13 January 2011, 4,213 mi^2^ of federal waters around the well and parts of area 12 in LA State coastal waters remained closed. On 1 February 2011, NOAA repoened the federal waters that had been reclosed to royal red shrimp fishing. On 19 April 2011, NOAA reopened all remaining federal waters. Approximately 1.5% of LA coastal waters remain closed.

The ecological and human health impacts of the DH oil blowout may differ from previous oil spills because of the depth at which the oil leak occurred, the large volume of oil released, and the unprecedented volume of dispersants (Corexit 9500 and Corexit 9527) used at the wellhead. Contamination of Gulf seafood has been a central concern for government agencies, fishing businesses, and consumers. An Institute of Medicine (IOM) report and a report from the Oil Spill Commission recommended continued analysis of Gulf seafood as an important component to determine potential long-term health impacts (IOM 2010) and restore consumer confidence in Gulf fisheries (Oil Spill Commission 2011). The goal of the present review is to inform risk assessment and monitoring efforts by *a*) synthesizing existing information on the duration and extent of seafood contamination after oil spills; *b*) evaluating the current regulatory process used to determine when it is appropriate to reopen fisheries in the Gulf; and *c*) providing recommendations for future testing and monitoring of seafood.

## Methods

We began by gathering information from scientific publications identified using web-based tools including Google Scholar (Google 2011), PubMed (National Center for Biotechnology Information 2011), and Web of Science (Thomson Reuters 2011), and combinations of the following search terms: “oil spill” (including individual names of previous and present oil spills), “seafood safety,” “heavy metals” (including individual metals), “polycyclic aromatic hydrocarbons” (PAHs; including specific individual PAHs), “fisheries,” “seafood consumption,” “finfish,” “shellfish,” “oysters,” “crustaceans,” “lobsters,” and “mollusks.” We also searched for these terms in federal and international organization technical reports and databases from NOAA, the FDA, the U.S. Environmental Protection Agency (EPA), the National Toxicology Program, the International Agency For Research on Cancer, and the World Health Organization (WHO). In addition, we consulted with government officials and academic experts involved in the development and implementation of the testing strategy for evaluating seafood safety after the DH oil spill.

## Results

*Seafood contamination after previous oil spills.* Studies after previous oil spills have shown that seafood contamination is determined by numerous factors, including the type and quality of the oil, the proximity of the spill to fishing grounds, ambient temperature and weather conditions, and species- and ecosystem-specific parameters that determine metabolism and the potential for bioaccumulation at different levels of the food chain. Most studies have focused on levels of PAHs in seafood after oil spills, and only a few have evaluated metal contamination. Very little is known about seafood contamination from dispersants, but toxicological studies of crude oil components and dispersants have been undertaken by international and national research bodies.

PAHs. PAHs are widespread organic pollutants containing two or more aromatic rings, and their toxicity varies widely among individual PAH compounds [summarized in Supplemental Material, Table 1 (doi:10.1289/ehp.1103507) for cancer end points and Supplemental Material, Table 2 for noncancer end points]. PAHs have been shown to accumulate in fish and shellfish, and concentrations of particular PAHs considered unsafe, particularly in shellfish, have been recorded after previous oil spills.

**Table 1:** Human health risk calculation parameters for reopening of fisheries waters after the DH blowout and previous U.S. oil spills.

Oil spill	Magnitude of spill (gallons)	Fisheries closures	RL*a*	BW (kg)	PAH CSF**(mg/kg/day)*b*	CR (g/day)	ED (years)	LOC (ppb BaPE)*c*	Reference
DH, Gulf of Mexico, 2010		206,000,000		1–12 months, area around platform opened 19 April 2011		1:100,000		80		7.3		Finfish: 49 Shrimp/crab: 13 Oysters: 12		5		Finfish: 35 Shrimp/crab: 132 Oysters: 143		NOAA/FDA 2010
T/V *Dubai Star*, San Francisco Bay, California, 2009		400–800		1 month		1:10,000		70		11.5		32.5 (one 8-ounce meal per week)		30		Fish and shellfish: 44		Klasing and Brodberg 2010
*Cosco Busan*, San Francisco Bay, California, 2007		58,000		1 month		1:10,000		70		11.5		32.5 (one 8-ounce meal per week)		30		Fish and shellfish: 44		Brodberg 2007
M/V *New Carissa*, Oregon, 1999		70,000		Bivalves: 21 days		1:1,000,000		70		7.3		Shellfish: 7.5 (average) 32.5 (high)		2		Shellfish: 10 (high) 45 (average)		Gilroy 2000
M/V *Kure*, California 1997		4,537		Oyster, crab: 49 days		1:1,000,000		70		9.5		Shellfish: 7.5 (average) 50 (high)		2		Shellfish: 5 (high) 34 (average)		Challenger and Mauseth 1998
T/V *Julie N*, Maine, 1996		180,000		Shellfish: 15 days		1:100,000		70		7.3		Lobster: 13.6		10, 30		Lobster: 50 (16) (for 30-year ED)		Mauseth et al. 1997
T/B *North Cape*, Rhode Island, 1996		828,000		Finfish and bivalves: 73 days Lobsters: 75–155 days		1:1,000,000		70		7.3		30		5		Lobster: 20		Mauseth et al. 1997
T/V *Braer*, Shetland Islands, 1993		25,000,000		Finfish: 2 months Farmed salmon: 2 years Lobster: > 6 years												Reach background levels of PAHs		Kingston 1999
T/V *Exxon Valdez*, Alaska*,* 1989		11,000,000		Herring/salmon: entire season Bivalves: advisories in four subsistence harvest areas		1:1,000,000		70		7.3		Salmon: 89 Other finfish: 52 Crustaceans: 21 Bivalves: 2		10, 70		Salmon: 3 (0.3) Finfish: 5 (0.5) Crustaceans: 11 (1.1) Bivalves: 120 (12) (for 70-year ED)		NOAA 2002
**a**Acceptable level of risk set for determining the LOC. **b**Rate of increase in cancer risk per unit dose. **c**Calculated LOC. See Equation 1 for further details.																		

**Table 2:** Estimated LOCs for children, women, and men, based on 1–2 seafood meals per week.

Young children (1–6 years of age)			Older children (7–12 years of age)			Adult women (≥ 20 years of age)			Adult men ( ≥ 20 years of age)		
Contaminant	2 meals/week	1 meal/week	2 meals/week	1 meal/week	2 meals/week	1 meal/week	2 meals/week	1 meal/week
PAH (BaPE) LOC (ppb)*a*		0.6–600		1–116		0.9–86		2–173		1–119		2–239		1–108		2–217
MeHg LOC (ppm)*b*		0.07		0.15		0.11		0.21		0.15		0.29		0.13		0.26
As (inorganic) LOC (ppm)*b,c*		0.0038–3.79		0.0074–7.36		0.0055–5.46		0.011–10.94		0.076–7.56		0.015–15.13		0.069–6.87		0.014–13.74
Cd LOC (ppm)*b*		0.73		1.46		1.05		2.1		1.46		2.9		1.32		2.64
DOSS LOC (ppm)*d*		72.84		145.70		105.05		210.11		145.56		291.12		132.10		264.20
MeHg, methylmercury. Body weights (portion sizes) for the populations were as follows: young children, 17.7 kg (3 oz); older children, 38.3 kg (4.5 oz); adult women, 70.7 kg (6 oz); adult men, 85.6 kg (8 oz). Body weights were set at the 50th percentile reported by McDowell et al. (2008). **a**Using a 1 in 1 million or 1 in 10,000 RL (range indicated), CalEPA CSF (11.5 mg/kg/day; CalEPA OEHHA 2005), and a 10-year ED. **b**Based on U.S. EPA IRIS RfDs (Cd, 0.001 mg/kg/day; MeHg, 0.0001 mg/kg/day) or CSF (As, 1.5 mg/kg/day) as reported by the U.S. EPA (2000) using the following formulas to calculate the LOCs: for Cd or MeHg, LOC = (*RfD*)(*BW*)(*CF*)/*CR*; for As, LOC = (*RL* × *BW* × *AT* × *CF*)/(*CSF* × *CR* × *ED*). **c**A small percentage (< 1–3%) of As found in fish and shellfish is inorganic As (Food Standards Agency 2005). **d**Based on FDA LOC (FDA 2010) use of the WHO/FAO ADI (0.1 mg/kg/day) (FAO/WHO 1995) and using the following formula to calculate the LOC: LOC = (*ADI*)(*BW*)(*CF*)/*CR*]. Note that the FDA uses a body weight of 60 kg instead of 80 kg (used for PAH LOC calculation) to calculate the DOSS LOC, based on WHO/FAO guidelines.																

Overall PAH levels provide an indication of the extent of potential contamination. However, because toxicity varies greatly among PAHs, information on individual PAH concentrations is needed to assess potential human health implications from these types of data. A toxic equivalency approach is often used to estimate risk based on the relative potency of individual PAHs relative to benzo[*a*]pyrene (BaP), but this approach does not take into account different modes of action, and toxic equivalency factors have been derived for only a small subset of PAHs found in oil (Reeves et al. 2001).

The duration of elevated PAH levels in seafood after previous oil spills has varied from several weeks to several years. High-molecular-weight PAHs (those with four or more benzene rings) are more likely to accumulate in tissues and persist for longer periods than low-molecular-weight PAHs (Meador et al. 1995). PAH levels after previous oil spills have been consistently lower in vertebrates compared with invertebrates (Law and Hellou 1999). This phenomenon is explained at least partly by the relatively well-developed cytochrome P-450 monooxygenase enzyme system in finfish (Hellou et al. 1995; Stegeman and Lech 1991), a system that results in increased metabolic clearance of PAH relative to bivalve mollusks, which have limited PAH metabolic capability, and crustaceans (Yender et al. 2002).

Several studies have reported evidence of increased PAH concentrations in seafood after oil spills compared with baseline levels. For example, the concentration of two- to six-ring PAHs in scallops after the *Braer* oil spill off the coast of the Shetland Islands was found to be > 1,300 ppb versus 12–90 ppb in reference samples (Kingston 1999); after the *Sea Empress* oil spill off the coast of Wales, concentrations ranged from 12 to 186 ppb in salmon samples compared with reference samples ranging from 9 to 86 ppb (Law et al. 1997) [Supplemental Material, Table 3 (doi:10.1289/ehp.1103507)]. Law et al. (2002) examined PAH levels in reference data sets and post–oil spill seafood across 19 studies  and found average total PAH concentrations between 20 and 1,600 ppb in baseline monitoring studies and between 104 and 27,400 ppb after various oil spills.

Time-series data sets from the *Sea Empress* oil spill showed that elevated PAH levels in most of the impacted area returned to baseline levels within 4 months in finfish and crustaceans, and within 6 months for bivalve mollusks (Law et al. 1997). PAH levels were increased in several species of fish and shellfish for 3 weeks after the *Braer* oil spill, but fell to baseline levels within 2 months in wild fish (Kingston 1999). However, salmon farms in the area were contaminated for 6 months, crabs and bivalves had elevated levels for 12 months, and the Norway lobster, which burrows in sediment, had elevated PAH levels for > 6 years (Kingston 1999). In contrast, PAH concentrations in mussels after the T/V *Dubai Star* oil spill in San Francisco Bay, California, fell to baseline levels after only 4 weeks (Klasing and Brodberg 2010). Findings based on the *Amoco Cadiz*, *Exxon Valdez*, *Braer*, and *Erika* oil spills suggest that contamination is particularly long-lasting when oil becomes entrained in sediment, where it can be remobilized by benthic organisms or storm events (Law et al. 2002). PAH levels in sediment and bivalves were elevated in many areas for approximately 3–4 years after the *Exxon Valdez* spill (Thomas et al. 1999), and PAH levels in mussels were elevated for 6 months after the much smaller 1997 M/V *Kuroshima* oil spill (NOAA 2002). Because of long-term PAH contamination, restrictions on oyster and lobster harvesting were in place for 7 years after the *Amoco Cadiz* and *Braer* oil spills (Law et al. 2002). Overall, studies of previous spills suggest that several factors may play a role in determining the duration of PAH contamination, including the amount of sedimentation and likelihood of subsequent resuspension of the oil, the composition of the oil, the rate of biodegradation (which tends to be higher in warmer climates), and the particular species of interest.

Metals. Metals are normal constituents of crude oil and drilling fluids used in the oil production process and bioaccumulation in seafood has been raised as a potential long-term health concern from the DH oil spill (Solomon and Janssen 2010). Metals including zinc (Zn), manganese (Mn), arsenic (As), cobalt (Co), chromium (Cr), selenium (Se), mercury (Hg), cadmium (Cd), copper (Cu), lead (Pb), nickel (Ni), tin (Sn), antimony (Sb), and vanadium (V) have been found to accumulate in sediments and marine organisms harvested from oil spill zones. In particular, Ni and V are found at significant levels in crude oil, and BP (2010) has reported concentrations of these elements ranging between 13 and 29 ppm for Ni and 10 and 106 ppm for V in Gulf of Mexico crude oils. In addition, Wainipee et al. (2010) recently suggested that there is potential for increased concentrations of As after an oil spill by creating a physical barrier and altering the chemistry of goethite, which normally binds As in ocean sediments; another study reported increased elemental Hg in air immediately after the MT *Hebei Spirit* oil spill off the coast of Korea (Pandey et al. 2009). As with PAHs, it is difficult to attribute metal contamination to a particular source (such as an oil spill), because metals also may accumulate in seafood as a result of normal geological phenomena such as ore formation, weathering of rocks, degassing, or leaching, or because of anthropogenic activities such as smelting, burning of fossil fuels, and discharges of industrial, agricultural, and domestic wastes (Osuji and Onojake 2004). However, good baseline data exist for concentrations of several metals in seafood from the Gulf of Mexico (Kimbrough et al. 2008; NOAA 2011; U.S. EPA 2009) that could be used to determine whether concentrations increased after the DH blowout.

Two studies looking at the occurrence and distribution of metals in mussels 2–4 years after the *Prestige* oil spill found that V was increased in post–oil spill samples when comparing with mussels from oiled with nonoiled sites and historical samples (Bartolomé et al. 2010; Villares et al. 2007). Another study conducted after the Jiyeh oil spill in the Eastern Mediterranean Sea found that heavy metals including Pb, Ni, and V were increased in oysters 305 days versus 72 days after the oil spill, whereas PAH levels had decreased (Barbour et al. 2009a) and levels had high positive correlation with size and length of the oysters (Barbour et al. 2009b). In a study of metal accumulation in marine blue crabs almost 10 years after the 1991 Gulf War oil spill, Al-Mohanna and Subrahmanyam (2001) found high concentrations of Zn and Cu in crabs at one sampling location and high concentrations of As, Pb, Mn, magnesium (Mg), Se, and V in crabs from another sampling site. Similarly, a study of the temporal variation and bioavailability of metals due to the oil industry in the Coatzacoalcos estuary in the southwestern Gulf of Mexico found average sediment metal concentrations that were highest for Zn, followed by Cu, Cd, and Pb (Ruelas-Inzunza et al. 2009). Results suggested that accumulation in sediment and marine organisms varied seasonally and that crabs and oysters bioaccumulated Cd, Zn, and Pb. In the Niger Delta, where several oil spills have occurred, Nduka et al. (2006) found high concentrations of metals including Pb, Zn, Cu, Ni, Cd, Co, Cr, Fe, and Mn in brains and gills of different freshwater and marine fish species.

Seafood tissue samples were not analyzed for metals after the *Cosco Busan* oil spill because levels of Cd, Cr, Cu, Pb, Zn, Hg, and Sn were below detection limits in samples of source oil; although Ni was detected, it was well below a level of health concern (Brodberg 2007). Seafood samples were analyzed for V after the T/V *Dubai Star* oil spill, but levels were reported to be well below the set health concern (43.75 ppm) and were not published (Klasing and Brodberg 2010).

In summary, previous studies indicate that metals present in oil can bioaccumulate in marine organisms and that higher levels of some metals have been documented after oil spills relative to baseline levels. In addition, contamination was noted 2 months after the Prestige oil spill (Villares et al. 2007) and may continue for > 10 years, as noted after the Gulf War oil spill (Al-Mohanna and Subrahmanyam 2001). However, the extent and persistence of seafood contamination with metals is likely to vary depending on the source oil, the type of organisms, and environmental conditions, and the lack of robust time-series data sets and inconsistent findings from previous studies make it difficult to predict the likelihood of metal contamination of seafood after the DH oil spill.

Dispersants. Dispersants are a mix of solvents and surfactants used to facilitate the breakup of oil into tiny droplets that are more easily broken down by natural processes. The two dispersants used in the DH oil spill—Corexit 9500 and 9527A—contain 2-butoxyethanol, propylene glycol, and dioctyl sodium sulfosuccinate (DOSS). Dispersants may offer significant protection to wildlife during a surface oil spill, but little is known about their effects after deepwater application, as used for the DH oil spill. Kujawinski et al. (2011) reported that DOSS underwent negligible biodegradation and was sequestered in deepwater hydrocarbon plumes 64 days after dispersant applications had ceased.

The approval of dispersants is contingent upon demonstrating that mortality in brown shrimp and mussels is not significantly greater in an oil–dispersant mixture than with an oil-only exposure (U.S. EPA 2010). Tests conducted by the U.S. EPA using the mysid shrimp, *Americamysis bahia*, and the inland silverside, *Menidia beryllina*—standard test organisms used in acute toxicity tests— revealed that the DH dispersants alone were less toxic than a dispersant–Louisiana sweet crude oil mixture, and that the toxicity of the oil–dispersant mixture was comparable with that of Louisiana sweet crude oil alone (Hemmer et al. 2010). Additional *in vitro* analyses suggested that cytotoxicity values of Corexit 9500 were indistinguishable from those of three other dispersants tested, but not used, during the DH spill, whereas two other dispersants on the market were significantly less cytotoxic (Judson et al. 2010).

*Role of regulatory agencies in seafood safety after the DH oil spill.* Responsibility for seafood safety management during and after the DH blowout is divided between state regulators and two federal agencies. In state waters, which extend from 0 to 3 miles offshore, responsibility lies primarily with state health agencies. Local fish consumption advisories or harvest closures are often issued based on water-quality monitoring programs, which may include analysis of finfish and shellfish tissues for contamination, and can be based on a variety of federal or state guidelines. The Food, Drug, and Cosmetic Act (2004) mandates that the FDA keep adulterated food off the market and gives the FDA jurisdiction over seafood in interstate commerce. Finally, the Magnuson-Stevens Fishery Conservation and Management Act (1996) gives NOAA the authority to regulate fishing in federal waters (3–200 miles from shore) and allows emergency action fisheries closures in the event of an oil spill.

It is important to stress that, after an oil spill, federal and state regulators must make rapid decisions, often based on limited data. Specifically, regulators must determine *a*) whether seafood harvesting in the spill area should be closed or restricted, *b*) what criteria should be met before reopening fisheries, and *c*) how to communicate these decisions and potential health risks to the public (Yender et al. 2002).

The first step of the decision-making process entails collection and evaluation of the characteristics, fate, and transport of the oil, and the identification of seafood resources at risk of exposure. If it is determined that oil will likely contaminate seafood, as was the case during the DH blowout, closures are issued. Here we focus on the criteria for reopening areas previously closed to fisheries, and hence, assessing seafood safety.

Method of testing. Decisions to reopen oil-impacted areas to seafood harvesting after the DH blowout are based on the NOAA/FDA protocol for the interpretation and use of sensory testing and analytical chemistry results (NOAA/FDA 2010). This protocol draws extensively from the NOAA publication “Managing Seafood Safety after an Oil Spill” (Yender et al. 2002), which details decision-making guidelines for state and federal regulators based on NOAA/FDA experiences during eight previous oil spills during 1989–1999.

Reopening criteria include an assessment based on monitoring for oil in harvest areas, collecting seafood samples from spill and reference areas, conducting sensory testing and/or chemical testing to determine whether seafood is unadulterated and fit for interstate commerce, and estimating the human health risk from consumption. Sensory testing, based on the smell of raw and cooked seafood samples, is conducted by a panel of 10 experts using the protocol outlined in a NOAA Technical Memorandum (Reilly and York 2001). Chemical analysis is performed using liquid chromatography (LC)/fluorescence detection or gas chromatography/mass spectrometry (GC/MS) to determine whether target PAH levels exceed the level of concern (LOC) set by the FDA risk assessment. Testing for metals is not part of the reopening protocol. Subsequent to reopening, NOAA developed and presented results of chemical analysis for DOSS in seafood tissue, which is a component of the dispersants used during the cleanup effort.

In general, many more samples have been assessed via sensory testing (i.e., the sniff test) than by chemical analysis. For example, 285 samples from finfish and 55 samples from shrimp taken from an area reopened on 15 November 2010 were assessed for contamination by sensory testing, whereas chemical analyses were performed on 33 composite samples of finfish (from a total of 207 fish) and 9 composite samples of shrimp (from 50 shrimp). We are unaware of studies examining the relationship between sensory testing and measured values of PAHs.

Sampling strategy. The NOAA Strategy for Future Reopenings web site (NOAA 2010d) indicates that the overall sampling scheme has been to work from the lesser-oiled outer boundaries of the originally closed areas toward the more heavily oiled areas immediately surrounding the DH wellhead, with larger numbers of samples collected from heavily oiled areas. For federal waters, closed areas were divided into 30-Nm^2^ grids, and at least six samples each of finfish, crab, and/or  shrimp were acquired from three to five sampling points within each grid. Composite samples formed by mixing several individual samples together are then tested for PAHs. Composite finfish samples may consist of one or two species or several species but include only fish found at the same depth (e.g., bottom feeders such as grouper would be grouped with other finfish species found at that depth) (NOAA, personal communication). Shellfish samples generally contain only one species. Requirements for sampling over time are not clear, but samples tested to support reopening of previously closed waters were collected within 2-day time frames prior to reopening, thus a time-series analysis was not conducted before reopening.

Determination of LOC. The LOC is the calculated concentration of chemical that, if found in seafood samples, would be considered unsafe for human consumption. The NOAA/FDA risk assessment for determining the LOC included a subset of 13 PAHs and their alkylated homologs [naphthalene, fluorene, anthracene, phenanthrene, pyrene, fluoranthene, chrysene, benzo[*b*]fluoranthene, benzo[*k*]fluoranthene, benz[*a*]anthracene, indeno[1,2,3-*cd*]pyrene, dibenz[*a,h*]anthracene, and BaP] based on known toxic potentials and previous oil spill risk assessments. PAH LOCs are determined using a cancer risk calculation based on benzo[*a*]pyrene equivalents (BaPE) for 7 PAHs [chrysene, benzo[*b*]fluoranthene, benzo[*k*]fluoranthene, benz[*a*]anthracene, indeno[1,2,3-*cd*]pyrene, dibenz[*a,h*]anthracene, and BaP]. The following equation is used to determine the LOC for carcinogenic PAH compounds:

LOC (BaPE) *=* (*RL × BW × AT × CF*)  ÷ (*CSF × CR × ED*). [1]

Parameters used to estimate the LOC for the DH spill and previous spills are shown in Table 1. For the DH spill, the risk level (*RL*) is set at 1 × 10^–5^; body weight (*BW*) is set at 80 kg based on average adult body weight (McDowell et al. 2008); averaging time (*AT*) is set at 78 years based on average life expectancy (Heron et al. 2009); the unit conversion factor (*CF*) is 1,000 µg/mg; the cancer slope factor (*CSF*) is set at 7.3 mg/kg/day based on the U.S. EPA BaP risk assessment for oral exposure (U.S. EPA 1994); and the seafood consumption rate (*CR*), which is set at 13 g/day  for shrimp and crab, 12 g/day for oysters, and 49 g/day for finfish, based on 90th percentile seafood consumers in 2005–2006 National Health and Nutrition Examination Survey (NHANES) study (CDC 2007). The exposure duration (*ED*) is 5 years, which is the estimated retention period of PAH contamination in seafood. The calculated LOCs for the DH spill are therefore 35 ng/g (ppb) BaPE for finfish, 132 ng/g (ppb) BaPE for shrimp/crab, and 143 ng/g (ppb) BaPE for oysters.

Determination of noncancer risk for the DH spill was based on U.S. EPA reference dose calculations (RfD; an estimate of a daily exposure of each chemical that likely has no significant risk during a lifetime) for the six additional PAHs (naphthalene, fluorene, anthracene, phenanthrene, pyrene, and fluoranthene):

LOC *=* (*RfD*)(*BW*)(*CF*) ÷ *CR.* [2]

*RfD*s used are from the U.S. EPA Integrated Risk Information System (IRIS): 0.02 mg/kg/day naphthalene, 0.04 mg/kg/day fluorene, 0.30 mg/kg/day anthracene/phenanthrene, 0.03 mg/kg/day pyrene, and 0.04 mg/kg/day fluoranthene). *BW*, *CF*, and *CR* are defined as above for the cancer risk assessment. Calculated LOCs range between 49.0 µg/g (pyrene in finfish) and 2,000 µg/g (anthracene/ phenanthrene in oysters). Note that these LOCs are much higher than those calculated for carcinogenic PAHs.

NOAA also developed and implemented a test to detect levels of DOSS, the dispersant component considered most likely to bioaccumulate, in seafood (NOAA 2010c). The acceptable daily intake (ADI) for DOSS is set at 0.1 mg/kg/day, based on the no observed adverse effect level of 50 mg/day for reproductive toxicity in rats and pulmonary circulatory effects in rabbits and dogs, with safety/uncertainty factors of 500 [Food and Agriculture Organization of the United Nations (FAO)/WHO 1995 ].

*Comparison of current protocol to seafood contamination analyses after previous oil spills.* Table 1 outlines fisheries closures and provides a comparison of the PAH cancer risk calculation parameters used for fisheries reopenings after previous oil spills; it also provides a context for evaluating the risk calculations used for the DH blowout. For example, the California Environmental Protection Agency (CalEPA) developed a protocol in the wake of the 2007 *Cosco Busan* and T/V *Dubai Star* oil spills [CalEPA Office of Environmental Health Hazard Assessment (OEHHA) 2007; Klasing and Brodberg 2010] that uses updated toxicity information based on another CalEPA risk assessment for PAHs, including an updated CSF and toxic equivalency factors for PAHs (CalEPA OEHHA 2005). The protocol (Klasing and Brodberg 2010) also takes into account noncancer end points for V. After the *New Carissa* oil spill, LOCs were determined based on the average shellfish consumer CR (45 ppb BaPE) and high-end CR (10 ppb BaPE) (Gilroy 2000). Shellfish was considered safe if analyses showed BaPE below the high-end consumer LOC (10 ppb BaPE).

As shown in Table 1, the LOCs calculated for the DH oil blowout are higher than for any previous oil spill risk assessment, particularly for shrimp and oysters, based on several factors. The T/V *Dubai Star* and *Cosco Busan* assessments used a 1 in 10,000 RL, whereas the T/V *Julie N* oil spill is consistent with the current NOAA/FDA protocol for the DH (NOAA/FDA 2010) using an RL of 1 in 100,000. Previous risk assessments, including those for the *Exxon Valdez*, have used an RL of 1 in 1 million. The RL sets the tolerable number of cancer cases attributable to the exposure. So, for example, an RL of 1 in 1 million means an exposure at the stated LOC is estimated to cause, at most, one additional cancer case in a population of 1 million people. All of these fall within the acceptable range of risks (1 × 10^–4^ to 1 × 10^–6^) used by the U.S. EPA in regulatory criteria for drinking water; 1 × 10^–4^ is provided as an example of a maximum acceptable RL in the U.S. EPA Guidance for Assessing Chemical Contaminant Data for Use in Fish Advisories (U.S. EPA 2000). The CalEPA OEHHA considered an RL of 1 × 10^–4^ appropriate for use in fish consumption advisories after the *Dubai Star* and *Cosco Busan* oil spills and as a counterbalance recognizing the health benefits of fish consumption (Brodberg 2007; Klasing and Brodberg 2010). In addition, the LOC for the DH blowout assumes a higher body weight (80 kg vs. the 70 kg used previously) based on recent NHANES data for average male weight (McDowell et al. 2008). However, in a recent survey of Gulf Coast residents, the Natural Resources Defense Council (NRDC 2010) reported that 60% of the adults weighed < 80 kg and 44% of the residents have children who eat seafood; this suggests that the U.S. average male weight may not be appropriate for predicting risk in all populations. Assumptions regarding seafood intakes also vary substantially among risk assessments. For example, the risk assessment for the *Exxon Valdez* spill specifically calculated risk based on subsistence levels of consumption seen in local populations (Fall et al. 1999), whereas other assessments used estimates for average and high-level consumers.  Although the DH consumption levels are based on a 90th percentile consumer, the CR for shellfish consumption (13 g/day for shrimp and crab and 12 g/day for oysters) is still well below estimates for high-end shellfish consumers used in recent risk assessments (*New Carissa*, *Cosco Busan*, and *Dubai Star* at 32.5 g/day and *Kure* at 50 g/day); a recent survey of Gulf residents (*n* = 547) suggests that shrimp CRs are actually 3.6–12.1  times higher than FDA estimates used for the DH risk assessment (NRDC 2010). The CalEPA used a more conservative CSF (11.5 vs. 7.3 mg/kg/day) based on recent toxicity analyses (CalEPA OEHHA 2005) in risk assessments for the *Kure*, *Cosco Busan*, and *Dubai Star* oil spills. ED estimates also vary considerably, with the *Exxon Valdez* assessment assuming a 10- or 70-year duration, the *Dubai Star* and *Cosco Busan* using 30 years, and the *New Carissa* and *Kure* assessments assuming an ED of 2 years.

*Federal and state seafood testing results from DH oil spill.* Federal seafood testing results released to date have demonstrated PAH levels at least two orders of magnitude below the LOCs established for the DH assessment (FDA 2010; NOAA 2010b). Supplemental Material, Table 3 (doi:10.1289/ehp.1103507) summarizes results in terms of total estimated PAH, when available, or BaPE, when available, to allow for comparison with previous studies, including studies after previous oil spills and studies of baseline levels. As mentioned above, estimated total PAH levels are not useful in determining risk to human health, because toxicity varies widely across specific PAHs; however, estimated total PAH levels do provide a useful metric for comparing across studies. Compared with estimated total PAH levels from previous spills, samples collected after the DH spill have PAH concentrations similar to or below levels reported in oyster, shrimp, and finfish samples after previous oil spills. However, this comparison can be made only with the current data released by the FDA (state water testing), as available NOAA data for federal waters do not include estimated total PAH levels. It is also of note that the seafood samples taken from coastal state waters were analyzed by the FDA using LC-fluorescence, and those from federal waters were analyzed by GC/MS according to the NOAA protocol (Sloan et al. 2004). In addition, when compared with studies looking at PAH concentrations from baseline samples around the world, samples collected after the DH spill have PAH levels within ranges found in these baseline monitoring data sets (see Supplemental Material, Table 3).

As of 9 January 2011, only 4 of 185 composite tissue samples acquired between June and September 2010 showed trace amounts of DOSS (2 tuna, 1 wahoo, and a mixed-species sample), all of which were far below the LOC value of 100 ppm for finfish and 500 ppm for shrimp, crabs, and oysters (NOAA 2010c). DOSS was detected in 6 of 18 red snapper and grouper composite samples collected at dockside between 8 July and 30 August at levels well below the LOC (mean, 0.12 ppm) (NOAA 2010c). One hundred twenty composite samples from state waters were also analyzed, and eight were found to have detectable levels of DOSS (six crab samples in Louisiana, one crab sample in Alabama, and one brown shrimp sample in Louisiana). Again, all were at least three orders of magnitude below the LOC.

## Discussion

We reviewed the protocol used for reopening fisheries and the results of seafood testing after the DH and previous oil spills to *a*) provide a foundation for recommendations on additional data collection and analysis that can facilitate the development of a more comprehensive assessment of seafood safety and *b*) to help restore consumer confidence in seafood from the Gulf of Mexico. We also make the following recommendations for addressing uncertainty and risk communication.

*1. Continue monitoring of PAHs.* Although levels of PAHs measured thus far in seafood after the DH blowout are well below levels that would be of concern for human health, continued monitoring should be in place to ensure that concentrations stay below LOCs, especially in light of potential recontamination from disturbance and redistribution of sedimented oil. Studies have shown significant sedimentation of the high-molecular-weight PAHs (Bouloubassi et al. 2005) that may be increased with the addition of dispersants in a coastal setting (Khelifa et al. 2008), whereas other reports suggest dispersants may decrease PAH sedimentation but increase PAH concentrations in the water column (Stakeniene and Jokšas 2004; Yamada et al. 2003). However, effects of large quantities of dispersant applied at considerable depths, as used after the DH oil spill, are unknown. Continued monitoring will ensure PAHs that may be present in sediment are not being redistributed and accumulating through the food chain.

*2. Begin testing for metals.* There is considerable uncertainty regarding the potential for increased metal contamination of seafood. In addition to trace levels of metals in oil, drilling fluids containing metals were also released into the Gulf after failed attempts to cap the oil well. Recent research suggests that oil spills may alter natural sedimentation processes of As, raising the concern that increased exposure via seafood consumption may be an issue (Wainipee et al. 2010). Even low-dose exposure to certain metals is of concern in pregnant women and children because of well-known impacts on neurodevelopment (Grandjean and Landrigan 2006).

We recommend additional testing focused on metals for which the U.S. EPA has developed risk-based consumption limits and for which there is reason for concern based on previous oil spills, including As, methylated Hg, and Cd, in addition to Ni and V, which are clearly associated with oil contamination and have known impacts on human health. In addition, based on information from previous oil spills, long-term monitoring over at least the next 5–7 years is necessary to ensure that any potential for bioaccumulation through the food chain is captured by the supplemental testing scheme.

*3. Continue monitoring of DOSS.* Although current evidence suggests minimal direct toxicity risk, there are still uncertainties regarding the long-term implications of the use of Corexit 9500 and Corexit 9527 dispersants. For example, it has been postulated that the unprecedented use of dispersants at the wellhead could increase bioaccumulation of oil components by increasing bioavailability and oil sedimentation, and a recent study confirmed that dispersants were found in deepwater plumes at least 2 months after the well was capped (Kujawinski et al. 2011).

Based on the NOAA dispersant tests of dockside seafood samples taken in July and August 2010, there is evidence of dispersant components in tissue, albeit at very low concentrations (NOAA 2010c). Continued monitoring of DOSS in finfish for at least 1 year is recommended, at which point monitoring could be implemented in areas where seafood with detectable levels of DOSS was previously harvested. Current information suggests that DOSS concentrations are unlikely to pose a risk to human health, but long-term monitoring is needed to determine whether DOSS persists in the Gulf ecosystem, particularly because this is the first time that large volumes of dispersants have been applied directly to a deepwater wellhead.

*4. Define and continuously reassess optimal short- and long-term sampling strategies.* Based on evidence of long-term contamination from previous oil spills, seafood monitoring should continue for several years. The frequency and spatial distribution of sample collection should be well defined and should include areas predicted to have exposure based on monitoring and modeling of subsurface plumes, as well as areas known to be oiled.

The full range of finfish and shellfish species should be sampled from all relevant sites using adequate sample sizes. The use of composite samples is necessary to limit costs, but the species included in composite samples should be clearly identified and communicated. Optimally, composite samples should only include one species from a single site, as diets, habitats, and behaviors vary widely, especially across finfish species.

Continuous reassessment of the sampling strategy based on testing results will ensure that efforts are focused on the most important contaminants, species, and areas. After an initial period of sampling the entire Gulf of Mexico, more focused and frequent sampling of hotspots (where detectable levels have been found in seafood and/or sediments) would then be appropriate.

*5. Develop guidelines to estimate variability and uncertainty in risk parameters.* As is evident from Table 1, estimated LOCs can vary widely based on underlying assumptions used in calculations. Therefore, we propose that guidelines be developed to estimate a range of LOC values according to age, sex, and seafood consumption parameters used after previous oil spills, as outlined below. An example of age-, sex-, and consumption- specific LOC ranges—assuming a 10-year ED, the 50th percentile body weight of a woman (70 kg) and average body weight of a 6-year-old child (22.68 kg), and one or two seafood meals per week—is shown in Table 2. We recommend the following:

- Define alternate LOCs based on a 1 in 1 million RL [traditionally used in U.S. EPA risk assessments and used as the risk LOC after the *Exxon Valdez* oil spill (NOAA 2002)] and 1 in 10,000 RL [used by CalEPA to take into account the health benefits of eating seafood (Klasing and Brodberg 2010)]. Resulting LOCs will differ by an order of magnitude.
- Estimate LOCs using EDs ranging from 5 to 30 years.
- Estimate LOCs across a variety of body weights appropriate for women and children.
- Estimate LOCs based on the CalEPA CSF (11.3 mg/kg/day) that uses updated toxicity parameters (Brodberg 2007).
- Estimate LOCs using a range of seafood eating patterns. Seafood consumption patterns vary widely across populations, but determining seafood consumption patterns in particularly vulnerable populations is critical for determining risk associated with seafood contamination. The current reliance on NHANES data sets may not adequately represent Gulf Coast populations, as suggested by a recent survey of Gulf residents (NRDC 2010). Additional survey work is necessary to define upper limits of seafood consumption along the Gulf Coast.

The lower ranges of PAH LOC values in Table 2, which are consistent with LOCs used for the *Exxon Valdez* and *Kure* oil spills (Table 1), suggest there is approximately an order of magnitude margin of safety based on FDA and NOAA chemical analyses of seafood after the DH oil spill. For example, concentrations of the individual carcinogenic PAHs tested to date have been below the limit of detection (LOD) (LODs between 0.07 and 0.28 ppb for individual PAHs across different BaPE values) and are well below the lowest LOC estimate for BaPE (0.60 ppb BaPE for young children who consume two seafood meals per week). Age- and consumption pattern–specific LOCs for methylmercury (MeHg), As, and Cd [based on U.S.EPA IRIS RfDs (U.S. EPA 2000)] and the dispersant component DOSS also are lowest for children, consistent with higher predicted exposures relative to body weight in children who eat fish frequently. This also highlights the importance of children as a vulnerable population.

*6. Communicate risk and uncertainty.* Since the reopening of one-third of previously closed Gulf waters to fisheries in late July 2010, numerous groups, including members of Congress, nongovernmental organizations, scientists, local fisherman, processors, and chefs, have raised concerns over the adequacy of the NOAA and FDA protocol for ensuring the safety of seafood caught in the Gulf. Issues raised include heavy reliance on an initial smell test, chemical testing for only a select number of PAH components of oil, small sample sizes for chemical analysis, the use of composite samples, and divergence from previous calculations used to determine the LOC for PAHs. Timely communication strategies would improve public understanding and trust in the federal process and should include the following.

Direct community engagement in the risk assessment process. Engagement should include discussion of current risk assessment and input on development of parameters used to calculate LOCs, as well as sensitivity and uncertainty analyses.

Timely and effective communication of testing results in the context of previous oil spills and baseline data. The presentation and use of baseline data, data from previous oil spills, and risks posed in relation to previous technological disasters and alternative routes of PAH exposure are critical to convey levels of risk in the context of previous disasters as well as in the context of other routes of exposure people may encounter. For example, PAH levels currently found in Gulf seafood are within ranges seen in routine surveillance data sets of seafood and a variety of other foodstuffs including meats and oils [Supplemental Material, Table 3 (doi:10.1289/ehp.1103507); Kim et al. 2008].

Presentation of testing results and sampling strategy in clear and simple formats. Using different formats to present testing results makes it difficult to convey overall trends in the data. Coordination of the presentation of results across federal and state agencies is critical for effective communication of seafood safety.

*7. Engage local communities in seafood safety testing.* Providing demonstrations and/or training for performing the FDA-approved smell test to interested community members at sentinel sites along the Gulf Coast may help to reassure consumers of the validity of the approach and create an efficient mechanism for ongoing monitoring, particularly at sites most heavily oiled or most in danger of being contaminated by other sources. In addition, continuous engagement of local community members in methods to determine seafood safety and making additional funding and technical guidance available to groups that want to conduct long-term chemical testing will provide a mechanism for timely and effective information exchange that may help to alleviate concerns over seafood safety after future disasters along the Gulf Coast.

*8. Increase knowledge base on toxicity of mixtures of contaminants.* Very little is known about the potential for interactions between various contaminants from an oil spill. For example, are effects of contaminants additive, or could they act synergistically? Alternatively, could effects of one contaminant reduce the impact of other contaminants? Mixtures or cumulative toxicity research is still in its infancy, but there is some evidence that concentration addition of toxicants impacting the same cellular pathway can predict toxicity of mixtures, at least in model systems (Syberg et al. 2008); a recent study suggested that synergism does exist for criteria air pollutants such as ozone (Mauderly and Samet 2009). For carcinogenicity of PAHs, the BaP equivalency is used to model additive effects across PAHs. Studies have shown neurodevelopmental impacts attributed to PAHs from combustion sources (Tang et al. 2006), and neurodevelopmental impacts are also related to some metals; therefore, interactions may exist based on similar end points of concern. Mixtures research of contaminants associated with oil spills should be a priority to more accurately define health risks after future oil spills.

## Conclusions

In the short term, a detailed monitoring and testing strategy that includes assessment of metals should be designed and coupled with an effective risk communication campaign to present the current results and long-term monitoring plans after the DH oil spill. Any decisions made based on the current protocol should be transparent, and inherent uncertainties should be fully discussed. We believe that implementation of these recommendations will help ensure the safety of seafood and restore consumer confidence in seafood from the Gulf Coast. In addition, a robust long-term monitoring and exposure assessment program is critical to improve our understanding of seafood safety after oil spills, which will provide invaluable information for greater preparedness in response to future oil spills.

## Supplemental Material

(296 KB) PDF
