# Supplementary material for: A Review of Seafood Safety after the Deepwater Horizon Blowout
Source: Environ Health Perspect. 2011 May 12;119(8):1062–9. doi: 10.1289/ehp.1103507 (PMC3237364; doi:10.1289/ehp.1103507)
Supplement: (296 KB) PDF [file ehp.1103507.s001.pdf]

SUPPLEMENTAL MATERIAL

A review of seafood safety after the Deepwater Horizon oil spill

Julia M Gohlke, Dzigbodi Doke, Meghan Tipre, Mark Leader, Timothy Fitzgerald

TABLE OF CONTENTS

Table 1: Toxicology of chemicals related to oil spills: Cancer Endpoints……………………………….*Page 3*

Table 2: Toxicology of chemicals related to oil spills: Non-cancer Endpoints………………………..*Page 7*

Table 3: Analytic testing in seafood following the *Deepwater Horizon* oil spill, previous oil spills, and other monitoring datasets ……………………………………………………………………………………….*Page 12*

References………*Page 14*

Supplemental Material, Table 1: Toxicology of chemicals related to oil spills: Cancer Endpoints

Chemical (CAS no.)	IARC Classification[1]	NTP Classification [2]	EPA Classification[3]	EPA IRIS RfD	Epidemiological Endpoint(s)/ and Route of Exposure	Animal/ Endpoint(s)/ /Route of Exposure	Comments
**PAHs**							
Naphthalene (91-20-3)	Group 2B	Part B		ORAL: 0.02 mg/kg-day INHALATION: 0.003 mg/m^3	Germ cell mutagen/inhalation, skin, ingestion	Rat/neuroblastomas of the olfactory epithelium and adenomas of the respiratory epithelium /inhalation.	Site specific metabolism [4]
Fluorene (86-73-7)	Group 3	Not Listed	Class D	ORAL: 0.04 mg/kg/day		Inadequate data [5]	Suspected gastrointestinal or liver toxicant/Ingestion skin contact (irritant), concern for osteosarcoma/ingestion[6]
Anthracene (120-12-7)	Group 3	Not Listed	Class D	ORAL: 0.3 mg/kg/day	Endocrine Toxicant, Gastrointestinal or Liver Toxicant, Skin or Sense Organ Toxicant		Included in the Substances of Very High Concern list (SVHC) by the European Chemicals Agency (ECHA) based on persistence, bioaccumulation and toxicity in freshwater and marine ecosystems.
Phenanthrene (CASRN: 85-01-8)	Group 3	Not Listed	Class D				Aquatic organism toxic ity[7]
Pyrene (129-00-0)	Group 3	Not Listed	Class D		Suspected: Neurotoxicant, Skin or Sense Organ Toxicant		
Fluoranthene (206-44-0)	Group 3	Not Listed	Class D	ORAL: 0.04 mg/kg/day	Suspected: Gastrointestinal or Liver Toxicant		Aquatic organism toxic ity[7]
Chrysene (218-01-9)	Group 2B	Not Listed	Class B2		inhalation, ingestion	Mice/Carcinomas and malignant lymphoma /intraperitoneal injection and skin carcinomas/dermal exposure. Hamsters and mice/chromosomal abnormalities germ/gavage exposure[8]	
benzo(b) fluoranthene (205-99-2)	Group 2B	Part B	Class B2		no human data	Mice/skin tumors /dermal and local sarcomas/injection and tumors following lung implant[8]	

Supplemental Material, Table 1: Toxicology of chemicals related to oil spills: Cancer Endpoints

Chemical (CAS no.)	IARC Classification[1]	NTP Classification [2]	EPA Classification[3]	EPA IRIS RfD	Epidemiological Endpoint(s)/ and Route of Exposure	Animal/ Endpoint(s)/ /Route of Exposure	Comments
benzo(k) fluoranthene (207-08-9)	Group 2B	Part B	Class B2		no human data and sufficient data from animal bioassays	Mice/tumors/lung implantation when administered with a promoting agent in skin-painting studies[8]	
benz(a) anthracene (56-55-3)	Group 2B	Part B	Class B2		no human data and sufficient data from animal bioassays.	Mice/tumors/gavage[8]	benz[a]anthracene is a component of mixtures that have been associated with human cancer.
indeno(1,2,3-cd)pyrene (193-39-5)	Group 2B	Part B	Class B2		no human data and sufficient data from animal bioassays	Mice/tumors/lung implants[8]	
dibenz(a,h) anthracene (53-70-3)	Group 2A	Part B	Class B2		no human data	Rat,guinea pigs, pigeons, fowl, adult and newborn mice/local sarcomas and lung adenomas[9]	
benzo(a) pyrene (50-32-8)	Group 1	Part B	Class B2		no human data	Mice/ skin tumors ,stomach tumors/ingestion, gavage, inhalation, dermal [8]	
**Dispersant components**							
2-butoxyethanol (111-76-2)	Group 3	Not listed		ORAL: 0.1 mg/kg-day INHALATION: 1.6 mg/m^3	Inhalation, ingestion,	Chronic Hemosiderin deposition in the liver/ Inhalation	No carcinogenicity studies on 2-butoxyethanol and 2-butoxyethanol acetate are available in people or animals. It is used as a solvent in spray lacquers, enamels, varnishes, and latex paints and as an ingredient in paint thinners and strippers, varnish removers, and herbicides. Has shown teratogenic effects in laboratory animals at high doses[12]
propylene glycol (57-55-6)	Not Listed	Not listed			Dermal, Inhalation, Ingestion		Generally recognized as safe in food and medications
dioctyl sodium sulfosuccinate (577-11-7)	Not Listed	Not Listed			Ingestion		ORAL (LD50): Acute: 1900 mg/kg [Rat]. 2643 mg/kg [Mouse][14] Not listed as a carcinogen by ACGIH, IARC, NTP, or CA Prop 65[15]
**Metals**							

Supplemental Material, Table 1: Toxicology of chemicals related to oil spills: Cancer Endpoints

Chemical (CAS no.)	IARC Classification[1]	NTP Classification [2]	EPA Classification[3]	EPA IRIS RfD	Epidemiological Endpoint(s)/ and Route of Exposure	Animal/ Endpoint(s)/ /Route of Exposure	Comments
Arsenic (7440-38-2)	Group 1	Part A		ORAL: 0.0003 mg/kg-day	skin, lung, digestive tract, liver, bladder, kidney, and lymphatic and hematopoietic systems	Rat and Mice/stomach adenocarcinoma, lymphocytic leukemia and lymphoma	
Mercury (7439-97-6)	Group 3	Not Listed	Class D	INHALATION: 0.0003 mg/kg-day			
Methylmercury (22967-92-6)	Group 2B	Not Listed	Class C	ORAL : 0.0001 mg/kg-day		Mice/renal adenomas, adenocarcinomas and carcinomas /ingestion	
Cadmium (7440-43-9)	Group 1	Part A		ORAL: 0.0005 mg/kg-day (water) 0.001 mg/kg-day (food)	lung, prostate-inhalation ingestion	Mice/lung tumors	
Vanadium (7440-62-2)	Not Listed	Not Listed				Rats and mice/alveolar/bronchiolar neoplasms/inhalation-Vanadium pentoxide[16]	Occurs only in chemically combined form. Vanadium pentoxide (1314-62-1) (IARC Group 2B) no studies specifically for cancer in humans[1] RfD: 9 x10^{-3} mg/kg-day
Nickel (7440-02-0)	Group 2B	Part A	Nickel refinery dust: Class A	ORAL: 0.02 mg/kg-day (soluble salts form)	lung and nasal cancer-inhalation	Rats and hamsters/tumors	
Aluminum (7429-90-5)	Group 1 (aluminum production)	Not Listed			lung, bladder, lymphosarcoma/reticulosarcoma, pancreatic - inhalation		Aluminum production (IARC Group1)[1]; cancer effects: none[12]
Lead (7439-92-1)	Group 2B	Part B	Class B2		digestive system, stomach, respiratory system, kidney, bladder - inhalation	Rats/renal tumors/ oral and subcutaneous	IARC: organolead compounds (Group 3)[1]

1 Agents Classified by the *IARC Monographs*, Volumes 1–100:
Group 1 Carcinogenic to humans
Group 2A Probably carcinogenic to humans
Group 2B Possibly carcinogenic to humans
Group 3 Not classifiable as to its carcinogenicity to humans
Group 4 Probably not carcinogenic to humans
http://monographs.iarc.fr/ENG/Classification/index.php

2 U.S. National Toxicology Program chemical classification:
A. Known to be a human carcinogen
B. Reasonably anticipated to be a human carcinogen
http://ntp.niehs.nih.gov/

3 EPA Cancer Classification
- Group A: "Human Carcinogen" There is enough evidence to conclude that it can cause cancer in humans.
- Group B1: "Probable Human Carcinogen" There is limited evidence that it can cause cancer in humans, but at present it is not conclusive.
- Group B2: "Probable Human Carcinogen" There is inadequate evidence that it can cause cancer in humans but at present it is far from conclusive.

- Group C: "Possible Human Carcinogen" There is limited evidence that it can cause cancer in animals in the absence of human data, but at present it is not conclusive.
- Group D: "Not Classifiable as to Human Carcinogenicity" There is no evidence at present that it causes cancer in humans.
- Group E: "Evidence of Non-Carcinogenicity for Humans" There is strong evidence that it does not cause cancer in humans.

http://www.greenfacts.org/glossary/def/epa-cancer-classification.htm#a1

4 Recent publications discussed clearly demonstrate that there are distinct differences in naphthalene metabolism in pulmonary and nasal tissues between primates and rodents. Collectively, papers show that primate lung had nasal tissue have very low metabolic activity as compared to rodents and the metabolism of naphthalene in target tissues is minimal, at best. The metabolic differences strongly suggest that primate and human lung and nasal tissues may not be susceptible to naphthalene induced cytotoxicity as a result of its site specific metabolism. http://www.naphthalenesymposium.org/Naphthalene%20Coalition%20Research%20Matrix.pdf

5 Faust et al. TOXICITY SUMMARY FOR FLUORENE. April 1994, http://cira.ornl.gov/documents/FLUORENE.pdf

6 ATSDR: Agency for Toxic Substances and Disease Registry. Minimal risk Levels for Hazardous Substances. January 2004. http://www.atsdr.cdc.gov/mrls.html

7 PAN Pesticides Database – Chemicals. Alphabetic list of all chemicals. http://www.pesticideinfo.org/List_ChemicalsAlpha.jsp

8 EPA Integrated Risk Information System (IRIS). http://www.epa.gov/ncea/iris/subst/0434.htm

9 Polycyclic Aromatic Hydrocarbons, 15 Listings. Substance Profiles, Report On Carcinogens, Eleventh Edition. http://ntp.niehs.nih.gov/ntp/roc/eleventh/profiles/s150pah.pdf

10 National Toxicology Program. NTP Study Reports. Toxicology and Carcinogenesis Studies of Toluene (CAS No.108-88-3) in F344/N Rats and B6C3F_1 Mice (Inhalation Studies) http://ntp.niehs.nih.gov/?objectid=0708DAFF-AAAC-9FD9-81F57FAA73DD574C

11 IARC Monographs on the Evaluation of Carcinogenic Risks to Humans. Musk Ambrette and Musk Xylene. 1996; 65,477-495

12 Agency for Toxic Substance Disease Registry. Toxic Substances Portal - 2-butoxyethanol. http://www.atsdr.cdc.gov/ http://www.atsdr.cdc.gov/toxfaqs/tf.asp?id=346&tid=61 http://www.atsdr.cdc.gov/substances/toxsubstance.asp?toxid=34

13 Material Safety Data Sheet. Mallinckrodt Baker, Inc. http://www.jtbaker.com/msds/englishhtml/b6100.htm

14 The Good Scents Company. dioctyl sodium sulfosuccinate. http://www.thegoodscentscompany.com/data/rw1240181.html

15 Global Bio-chem MSDS Report. Biopropylene glycol. http://www.globalbiochemna.com/uploads/BioPG_MSDS.pdf

16 IARC Monographs on the Evaluation of Carcinogenic Risks to Humans. Vanadium Pentoxide. 2006;86;page 227-292 http://monographs.iarc.fr/ENG/Monographs/vol86/mono86-10.pdf

Supplemental Material, Table 2: Toxicology of chemicals related to oil spills: Non-cancer Endpoints

Chemical (CAS no.)	Endpoint(s) evaluated	Route of exposure	Findings	EPA IRIS RfD	FDA findings	Comments
**PAHs**						
naphthalene (91-20-3)	chronic haemolytic anaemia, cataracts,	inhalation, ingestion		ORAL: 0.02 mg/kg-day INHALATION: 0.003 mg/m^3	Study used a mixture of 8 PAH's- at low (7.57 ug/l) and high concentration (72.3 ug/L). Salinity 34 ppt. Concentrations generally peaked and reached a steady state btwn 2-7d of the 36 d exposure, at approx=10,000 ug/g. K2=1.3/d BCF=895[1]	Used in mothballs and toilet bowl blocks
fluorene (86-73-7)	Dermal, musculoskeletal, ocular, respiratory. Decreased RBC, packed cell volume and hemoglobin - Mouse subchronic study	oral, inhalation	hypoactivity, decrease in red blood cell count and packed cell volume, decreasing trend in BUN and a significant increasing trend in total serum bilirubin, increase liver weight/mice [2]	ORAL: 0.04 mg/kg-day		frequently detected in the vapor phase of various PAH emission sources, including coal tar pitch, petroleum refineries, diesel exhaust fumes, and tobacco smoke, where it is the second most abundant PAH [3]
anthracene (120-12-7)	No observed subchronic toxicity effects-study in mice	oral gavage	reduced survival[4]	ORAL: 0.3 mg/kg-day	No observed effects	Because PAH chemicals occur in mixtures, potential health effects must be inferred [4]
phenanthrene (85-01-8)	Suspected respiratory, skin and sense organ toxicant (NTP-HS) [5]	oral, dermal	reproductive, body weight, skin/mice			
pyrene (129-00-0)	Suspected neurotoxicant, skin or sense organ toxicant [5] Kidney effects (renal tubular pathology, decreased kidney weights)-mouse subchronic oral bioassay	oral		ORAL: 0.03 mg/kg-day	Kidney effects (renaltubular pathology, decreased kidney weights)[6]	
fluoranthene (206-44-0)	nephropathy, increased liver weights, hema-tological alterations, and clinical effects-Mouse subchronic study [6]	ingestion		ORAL: 0.04 mg/kg-day		

Supplemental Material, Table 2: Toxicology of chemicals related to oil spills: Non-cancer Endpoints

Chemical (CAS no.)	Endpoint(s) evaluated	Route of exposure	Findings	EPA IRIS RfD	FDA findings	Comments
chrysene (218-01-9)		inhalation			cardiovascular toxicant, reproductive or developmental toxicant [7]	
benzo(b)fluoranthene (205-99-2)		ingestion, inhalation			cardiovascular toxicant [7]	
benzo(k)fluoranthene (207-08-9)		ingestion, inhalation			cardiovascular toxicant [7]	
benz(a)anthracene (56-55-3)		ingestion, inhalation			cardiovascular toxicant [7]	
indeno(1,2,3-cd)pyrene (193-39-5)		ingestion, inhalation				
dibenz(a,h)anthracene (53-70-3)		ingestion, inhalation			reproductive or developmental toxicant [7]	
benzo(a)pyrene (50-32-8)		ingestion, inhalation	mutagen, carcinogen		cardiovascular toxicant, reproductive or developmental toxicant [7]	
**Dispersant components**						
2-butoxyethanol (111-76-2)	Rats and mice/ hematologic impacts, stomach ulcers, epithelial hyperplasia	inhalation	Oral rat LD50: 470 mg/kg; Inhalation rat LC50: 450ppm/4H; Skin rabbit LD50: 220 mg/kg; Reproductive Toxicity: Has shown teratogenic effects in laboratory animals.	ORAL: 0.1 mg/kg-day INHALATION: 1.6 mg/m^3	Listed under Everything Added to Food in the United States (EAFUS) regnums: 173.315, 175.105, 176.210, 177.1650, 178.1010 [9]	Prolonged or repeated exposures can cause damage to the liver, kidneys, lymphoid system, blood and blood-forming organs.
propylene glycol (CASRN: 57-55-6)	At lethal or near lethal doses (6 g per kg or more), it has been reported to cause kidney damage in several species and toe deformities in chicks.	ingestion	Toxicity generally occurs only at plasma concentrations over 1 g/L, which requires extremely high intake over a relatively short period of time.	reviewed by the U.S. EPA RfD/RfC Work Group and determined to be inadequate for the derivation of an inhalation RfC	Database of Select Committee on GRAS Substances (SCOGS) Reviews - There is no evidence in the available information on propylene glycol and propylene glycol monostearate that suggesets a hazard to the public when they are used at levels that are now current or that might reasonably be expected in future.	

Supplemental Material, Table 2: Toxicology of chemicals related to oil spills: Non-cancer Endpoints

Chemical (CAS no.)	Endpoint(s) evaluated	Route of exposure	Findings	EPA IRIS RfD	FDA findings	Comments
dioctyl sodium sulfosuccinate (577-11-7)	Rats/decline in growth rate in rats, complete mortality at 4% or 8% in the feed. A three-generation reproduction study with DSS in rats did not reveal any adverse effects on the reproductive function of either sex at dose levels up to 10 g/kg in the diet.	ingestion	Skin and respiratory irritant. Severe eye irritant.		Acceptable Daily Intake (ADI) of 6 mg/person/day	A common ingredient in consumer products, especially laxatives of the stool softeners. ORAL (LD50): Acute: 1900 mg/kg [Rat]. 2643 mg/kg [Mouse].
**Metals**						
arsenic (7440-38-2)	Human/Hyperpigmentation, keratosis and possible vascular complications;	ingestion		ORAL: 0.003 mg/kg/day		
mercury (7439-97-6) and methylmercury	Developmental neurotoxicant, Hand tremor, memory disturbance;	inhalation, ingestion	The nervous system is very sensitive to all forms of mercury. Methylmercury and metallic mercury vapors are more harmful than other forms.	ORAL: 0.0001 mg/kg/day INHALATION: 0.003 mg/m^3		
cadmium (7440-43-9)	emphysema, chronic obstructive pulmonary disease (COPD) in smokers. Development of peripheral artery disease. Progressive renal tubular dysfunction. Reduced mineral density in bone. Increased rates of preterm delivery.	inhalation, ingestion	Affected Organ Systems: Cardiovascular (Heart and Blood Vessels), Developmental (effects during periods when organs are developing) , Gastrointestinal (Digestive), Neurological (Nervous System), Renal (Urinary System or Kidneys), Reproductive (Producing Children), Respiratory (From the Nose to the Lungs)	ORAL: 0.0005 mg/kg/day (water) 0.001 mg/kg/day (food)	Maximum limit of cadmium in bottled water: 0.005 mg/L.	
vanadium (7440-62-2)	Rats/Histological changes in kidneys, lungs, and the spleen.	inhalation, ingestion	Vanadium pentoxide (V2O5)[CASRN: 1314-62-1] V2O5 can pass the blood–placenta barrier and is teratogenic in rodents. Eye, skin and respiratory tract irritation have been associated with human exposure to elemental vanadium and V2O5.	ORAL: 0.009 mg/kg/day for Vanadium pentoxide[6] ATSDR has derived an acute-duration inhalation MRL of 0.0008 mg vanadium/ m^3 [(10)]		Most people are exposed daily to very low concentrations of vanadium in food, drinking water, and air. The vanadium in these sources is at least partially due to naturally occurring vanadium.

Supplemental Material, Table 2: Toxicology of chemicals related to oil spills: Non-cancer Endpoints

Chemical (CAS no.)	Endpoint(s) evaluated	Route of exposure	Findings	EPA IRIS RfD	FDA findings	Comments
nickel (7440-02-0) nickel, soluble salts (CASRN various)	Decreased body and organ weights in rats. Affected Organ Systems: Cardiovascular (Heart and Blood Vessels), Dermal (Skin), Immunological (Immune System), Respiratory (From the Nose to the Lungs).	Dermal, inhalation, ingestion	Lung effects including chronic bronchitis and reduced lung function have been observed in workers breathing nickel.	ORAL: 0.02 mg/kg/day (Nickel, soluble salts)	There is no evidence in the available information on elemental nickel that suggests a hazard to the public when it is used at levels that are now current and in the manner now practiced or that might reasonably be expected in the future.	The most common reaction is a skin rash at the site of contact.
aluminum (7429-90-5) aluminum phosphide (20859-73-8)	Increase in alveolar macrophages; granulomatous lesions, increase lung weight in lung in guinea pig and rat. decreased pup body weight;	Dermal, inhalation, ingestion	Several deaths have been reported after occupational exposure to a finely powdered metallic aluminum.	ORAL: 0.0004 mg/kg/day for aluminum phosphide	Aluminum and its salts are found in varying amounts in nearly all foods. It has been estimated that the daily aluminum intake from all dietary sources ranges from 10 to 100 mg per day.	Neurological and skeletal effects have been observed in children with impaired renal function after abnormal accumulation of aluminum due to exposure to aluminum-contaminated dialysate [11]
lead (7439-92-1)	Neurodevelopmental impacts, decreased sperm count in men and spontaneous abortions in women, neurotoxicity, hypertension, impaired hearing acuity, impaired hemoglobin synthesis,	ingestion, inhalation	Immediately Dangerous to Life and Health (IDLH) = 100 mg/m^3 (as metallic lead) Recommended Exposure Limit (REL) = 0.050 mg/m^3 (metallic lead, lead oxides, and lead salts (including organic salts such as lead soaps but excluding lead arsenate)).	Blood lead levels of 10 ug/dL [12]	Food: cannot exceed concentrations which range from 0.1-10 ppm. Ceramics: from 0.5-3.0 ug/mL of leach solution. Maximum permissible level of lead in bottled water = 0.005 mg/L	FDA has established Provisional Daily Total Tolerable Intakes (PDTTI) for lead for several at-risk groups.

1 FDA Phish-Pharm Listing. Bioconcentration, biotransformation, and elimination of polycyclic aromatic hydrocarbons in sheepshead minnows (Cyprinodon variegatus) exposed to contaminated seawater. http://www.accessdata.fda.gov/scripts/fcn/fcnDetailNavigation.cfm?rpt=phishPharmListing&id=1325

2 Toxicity Summary for Fluorene. Oak Ridge National Laboratory 1994. http://cira.ornl.gov/documents/FLUORENE.pdf

3 Centers for Disease Control and Prevention. *National Report on Human Exposure to Environmental Chemicals: Chemical Information.* Fluorene. http://www.cdc.gov/exposurereport/data_tables/Fluorene_ChemicalInformation.html

4 Agency for Toxic Substances and Disease Registry (ATSDR). Toxicological Profile For Polycyclic Aromatic Hydrocarbons**.** U.S. Department of Health and Human Services. http://www.atsdr.cdc.gov/toxprofiles/tp69.pdf

5 Scorecard The Pollution Information Site. http://www.scorecard.org/chemical-profiles/summary.tcl?edf_substance_id=85-01-8

6 U. S. Environmental Protection Agency Integrated Risk Information System. http://www.epa.gov/IRIS/subst/0445.htm

7 FDA Draft Initial List of Harmful/Potentially Harmful Constituents in Tobacco Smoke or Smokeless Tobacco Products July 2010. http://www.fda.gov/downloads/AdvisoryCommittees/CommitteesMeetingMaterials/TobaccoProductsScientificAdvisoryCommittee/UCM219548.pdf

8 Tobacco Products Constituents Subcommittee. Draft Initial H/PH Constituents and their Association with Selected Tobacco Product-Related Diseases July 2010. http://www.fda.gov/downloads/AdvisoryCommittees/CommitteesMeetingMaterials/TobaccoProductsScientificAdvisoryCommittee/UCM217519.pdf

9 FDA List of Indirect Additives used in Food Contact Substances 2010. http://www.accessdata.fda.gov/scripts/fcn/fcnNavigation.cfm?rpt=iaListing&page=32

10 ATSDR Toxicological Profile for Vanadium 2009. http://www.atsdr.cdc.gov/toxprofiles/tp58-c8.pdf

11 U. S. Department of Health and Human Services Agency for Toxic Substances and Disease Registry. Toxicological Profile for Aluminum. Syracuse Research Corp. July 1999.

12 U.S. Centers for Disease Control and Prevention http://www.cdc.gov/nceh/lead/policy/changeBLL.htm

NOTES:

Very little epidemiological information is available on the individual chemicals within the PAH group. Most of the information available is for the PAH group as a whole. ATSDR did not derive any inhalation minimal risk levels (MRLs) for PAHs because no adequate dose-response data that identify threshold levels for non-cancer health effects are available in humans or animals for any duration of exposure. Animal studies showed that exposing mice to 308 parts per million (ppm) of PAHs (specifically benzo (a) pyrene) in food for 10 days (short term exposure) caused birth defects. Mice exposed to 923 ppm of benzo (a) pyrene in food for months developed problems in the liver and blood.
http://www.epa.gov/osw/hazard/wastemin/minimize/factshts/anthrace.pdf

Supplemental Material, Table 3. Analytic testing in seafood following the Deepwater Horizon blowout, previous oil spills, and other monitoring datasets

Event	Location	Species	Number of samples (most are composites)	Est. Total PAHs mean (± SD or range) in ppb wet weight	BaP Equivalent	Reference
Deepwater Horizon						
FDA testing	Gulf of Mexico-State waters	Finfish<br>Red Snapper<br>Grouper	N= 114<br>N=7<br>N=1	143 (265)<br>21 (28)<br>28	Not reported, but all individual PAHs far below LOCs	(FDA 2010)
		Shrimp	N=70	56.9(79.4)		
		Crab	N=67	411(469)		
		oyster	N=36	3676 (1710)		
NOAA testing	Gulf of Mexico-Federal waters	Finfish<br>Red Snapper<br>Grouper	N=206<br>N=6<br>N=8	Not reported	Not reported, but all individual PAHs far below LOCs	(NOAA 2010)
		Shrimp	N=56	Not reported	Not reported	
Independent tests						
LEAN	Lower Miss.	Oysters, mussels, crab, shrimp	N=9	16-386	Not reported	(LEAN 2010)
Previous oil spills						
T/V Dubai Star	San Francisco Bay	Mussels	N=22 (470 individual mussels)	Not reported	15.5 (Week 1)<br>34.7, 17.7 (Week 3)<br>3.0, 4.3 (Week 4)	(Klasing and Brodberg 2010)
		Clams	N=5 (166 individual clams)	Not reported	6.6 (Week 0)<br>2.8 (Week 1)	
Cosco Busan	San Francisco Bay	Finfish	N=7 composites	Not reported	< LOD	(Brodberg 2007)
		Crabs	N=16 composites	Not reported	0.65	
		Mussels	N=15 composites	Not reported	11-53	
M/V Kuroshima	Alaska	Mussels	Not reported	74,750; 953 (6 months after)	Not reported	(NOAA 2002)
Braer	Brittany	lobsters	Not reported	980	Not reported	(Kingston 1999)
		crabs	Not reported	2300	Not reported	

		scallops	Not reported	1300	Not reported	
Sea Empress	Wales	Salmon	Not reported	12 to 186	Not reported	(Law et al. 1997)
		mussel	Not reported	34 to 19,500	Not reported	
Exxon Valdez	Alaska	seafood	N=42<br>N=2297	0 to 18,460<br>0 to 41,840	Not reported	(Fall et al. 2001)
Tetney monobuoy	England		N=23	214 to 322	Not reported	(Law et al. 2002)
Erimo	England		N=23	235 to 631	Not reported	(Law et al. 2002)
Lagik	England		N=23	143 to 355	Not reported	(Law et al. 2002)
Background survey and monitoring datasets						
Mussel Watch	USA	mussel	N=1689	700(1.0 to 3760)	Not reported	(Kimbrough et al. 2008)
	England and Wales	shellfish	N=85	36 to 8930	Not reported	(Law et al. 2002)
	Arcachon Bay, France	mussel	Not reported	39 to 338	Not reported	(Law et al. 2002)
Sea Empress (Ref. samples)	Wales	salmon	Not reported	9 to 86	Not reported	(Law et al. 1997)
Braer (Ref. Samples)		scallops	Not reported	12 to 290	Not reported	(Kingston 1999)
	Catalonia, Spain	clam	Not reported	21.5	Not reported	(Llobet et al. 2006)
		mussel		22.4		
		shrimp		15.9		
		sardine		5.3		
		tuna		4.0		
		swordfish		6.0		
		Red mullet		3.1		
	Persian Gulf	Oysters, clams, scallops	N=9	32.65 (6.6 to 154)*	Not reported	(Tolosa et al. 2005)
	Gulf of Naples, Italy	mussel	Not reported	334 (105 to 831)	Not reported	(Amodio-Cocchieri et al. 2003)
	European markets	clams	Not reported	12.3(2.1 to 24.5)	Not reported	(Binelli and Provini 2003)
	Catalonia, Spain	Seafood (avg)	N=18	2.84 (14.34 in mussel samples)	Not reported	(Martorell et al. 2010)

*used dry weight to wet weight conversion factor of 0.18 based on CRESP document

REFERENCES

Amodio-Cocchieri, R., S. Amoroso, A. Arnese, T. Cirillo, P. Montuori & M. Triassi (2003) Pollution by mercury, arsenic, lead, chromium, cadmium, and polycyclic aromatic hydrocarbons of fish and mussels from the Gulf of Naples, Italy. *Bull Environ Contam Toxicol,* 71**,** 551-60.

Binelli, A. & A. Provini (2003) POPs in edible clams from different Italian and European markets and possible human health risk. *Mar Pollut Bull,* 46**,** 879-86.

Brodberg, R. 2007. Report on the safety of consuming fish and shellfish from areas impacted by the *M/V Cosco Busan* oil spill in San Francisco Bay, California. ed. C. E. P. Agency. Sacramento.

Fall, J. A., R. Miraglia, W. Simeone, C. J. Utermohle & R. J. Wolfe. 2001. Long-term consequences of the Exxon Valdez oil spill for coastal communities of southcentral Alaska. ed. A. D. o. F. a. Game. Juneau, AK.

FDA. 2010. Gulf of Mexico Oil Spill Update. http://www.fda.gov/Food/FoodSafety/Product-SpecificInformation/Seafood/ucm210970.htm#background_testing.

Kimbrough, K. L., W. E. Johnson, G. G. Lauenstein, J. D. Christensen & D. A. Apeti. 2008. An assessment of two decades of contaminant monitoring in the nation's coastal zone. 105 pp. Silver Spring, MD.

Kingston, P. 1999. Recovery of the marine environment following the Braer spill, Shetland. In *Proceedings 1999 Oil Spill Conference*, 103-109. Seattle, WA.

Klasing, S. & R. Brodberg. 2010. Report on the safety of consuming fish and shellfish from areas impacted by the T/V Dubai Star oil spill in San Francisco Bay, California. ed. C. E. P. Agency. Sacramento.

Law, R. J., C. Kelly, K. Baker, J. Jones, A. D. McIntosh & C. F. Moffat (2002) Toxic equivalency factors for PAH and their applicability in shellfish pollution monitoring studies. *Journal of Environmental Monitoring,* 4**,** 383-388.

Law, R. J., C. A. Kelly, K. L. Graham & R. J. Woodhead (1997) Hydrocarbons and PAH in fish and shellfish from southwest Wales following the *Sea Empress* oil spill in 1996. *1997 International Oil Spill Conference***,** 205-211.

LEAN (2010) Louisiana Environmental Action Network independent testing results http://leanweb.org/news/latest/bp-oil-spill-seafood-sampling-project-results-overview.html [accessed Dec. 15th 2010]

Llobet, J. M., G. Falco, A. Bocio & J. L. Domingo (2006) Exposure to polycyclic aromatic hydrocarbons through consumption of edible marine species in Catalonia, Spain. *J Food Prot,* 69**,** 2493-9.

Martorell, I., G. Perello, R. Marti-Cid, V. Castell, J. M. Llobet & J. L. Domingo (2010) Polycyclic aromatic hydrocarbons (PAH) in foods and estimated PAH intake by the population of Catalonia, Spain: Temporal trend. *Environ Int,* 36**,** 424-32.

NOAA. 2002. Restoration Plan and Environmental Assessment for the *M/V Kuroshima* oil spill, Summer Bay, Unalaska, Alaska. ed. r. Damage assessment, and restoration program. Alaska: http://www.darrp.noaa.gov/northwest/kuro/admin.html.

---. 2010. Deepwater Horizon/BP oil spill: Federal fisheries closure and other information. ed. http://sero.nmfs.noaa.gov/deepwater_horizon_oil_spill.htm. NOAA Fisheries Service.

Tolosa, I., S. J. de Mora, S. W. Fowler, J. P. Villeneuve, J. Bartocci & C. Cattini (2005) Aliphatic and aromatic hydrocarbons in marine biota and coastal sediments from the Gulf and the Gulf of Oman. *Mar Pollut Bull,* 50**,** 1619-33.
